# Dental Evaluation Prior to Cancer Therapy

**DOI:** 10.3389/froh.2022.876941

**Published:** 2022-04-18

**Authors:** Chee Weng Yong, Andrew Robinson, Catherine Hong

**Affiliations:** ^1^Discipline of Oral and Maxillofacial Surgery, Faculty of Dentistry, National University of Singapore, Singapore, Singapore; ^2^Discipline of Orthodontics and Paediatric Dentistry, Faculty of Dentistry, National University of Singapore, Singapore, Singapore

**Keywords:** radiotherapy, antineoplastic agents, bone density conservation agents, dental care, dental service, hospital

## Abstract

A comprehensive oral examination and dental care prior to the start of cancer therapy is the standard of care in many cancer centers. This is because good oral health will likely minimize the undesirable complications such as opportunistic infections during cancer therapy. As the considerations differ between anti-neoplastic regimens, this chapter discusses the indications and rationale when planning and executing a treatment plan for patients undergoing various cancer therapies.

## Introduction

Many cancer centers routinely recommend that patients undergo a comprehensive oral examination and if necessary, receive dental treatment prior to the commencement of anti-neoplastic therapy [1, 2]. This concept is commonly referred to as “dental clearance” and the rationale for this is based on the assumption that good oral health can likely minimize the undesirable oral sequelae from anti-neoplastic therapy [3, 4]. For instance, basic oral care strategies to improve oral health modifies the oral microbial load, which is proposed to decrease oral mucositis severity via modulation of the host inflammatory response [5]. As there are ethical issues with the conduct of clinical trials evaluating the benefit of dental clearance, the evidence regarding the effectiveness of dental clearance protocols and the extent of clearance needed to prevent or minimize oral complications arising during anti-neoplastic therapy is limited [2]. Thus, dental clearance protocols often vary between cancer centers; especially with regard to the aggressiveness of dental therapy needed (e.g., need for removal of asymptomatic partially impacted third molars). Despite these differences, the majority of dental clearance protocols generally follow these principles: [1] to stabilize and/or remove existing and potential sources of infection and irritation in the oral cavity and [2] to educate patient regarding the acute and chronic oral manifestations associated with anti-neoplastic therapy as well as oral care recommendations throughout their anti-neoplastic therapy [1, 4].

This aim of this chapter is to review the indications, rationale and guiding principles when planning and executing a dental treatment plan for patients undergoing anti-neoplastic therapy.

## Indications

As the considerations differ between anti-neoplastic regimens, dental practitioners should be cognizant of the rationale and objectives for dental clearance for the various anti-neoplastic therapies.

### Anti-neoplastic Chemotherapy and Hematopoietic Stem Cell Transplantation

The main mechanism of anti-neoplastic chemotherapeutics is the inhibition of cell proliferation and growth [6]. The majority of these agents do not differentiate between the cancer and healthy tissues; thus rapidly dividing non-cancerous tissues such as the hair follicles, skin or the bone marrow are also affected by anti-neoplastic chemotherapeutics [7]. Of significance to dentistry is the suppression of the bone marrow resulting in immunosuppression, which predisposes the patient to increased risk of opportunistic viral and fungal infections [8, 9]. Exacerbation of pre-existing oral or dental infections can also occur and may be complicated by superinfection and necrosis [10–13]. Oral mucositis, which is associated with the use of certain chemotherapy agents (e.g., methotrexate, doxorubicin, 5-fluorouracil, busulfan, bleomycin, and platinum co-ordination complexes) further increases the risk of a systemic infection from a local site due to the loss of an intact oral mucosal barrier [13–17].

The extent of bone marrow suppression is dependent on the chemotherapeutic regimen. Non-myeloablative regimens are reduced in intensity and do not completely suppress the bone marrow. They are usually used as adjuvant treatment for a variety of solid organ malignancies. High-dose myeloablative chemotherapy regimens are typically indicated for patients with hematological malignancies and are associated with a significant decline in hemoglobin, platelet and neutrophil levels. This occurs about 7 days after the drug administration, with the nadir occurring between 10 and 14 days and recovery in 3–4 weeks. The recovery to functional blood count levels is prolonged in some patients for various reasons such as advanced age, decreased clearance of chemotherapeutic drugs due to renal or liver dysfunction or concurrent radiotherapy to the bone marrow [18–20]. For allogeneic hematopoietic stem cell transplantation recipients, a certain degree of immunosuppression is deliberately maintained for 6–12 months after myeloablative chemotherapy for prophylaxis against graft-vs.-host-disease [21]. For the reasons mentioned above, the primary aim for dental evaluation in patients undergoing anti-neoplastic chemotherapy and hematopoietic stem cell transplantation is to prevent and minimize the occurrence of opportunistic infections and the potential systemic spread of a local infection [22, 23].

### Head and Neck Radiation Therapy

Radiation therapy is the use of ionizing radiation to diminish or kill cancer cells. Unlike anti-neoplastic chemotherapy where only rapidly proliferating cells are targeted, radiation therapy affects all structures in the exposed field. The main dental concern with head and neck radiation therapy (HNRT) is the life-long risk of Osteoradionecrosis of the Jaw (ORNJ) development with radiation doses ≥ 60 Gy [24–32]. ORNJ is defined as a slow-healing radiation-induced ischemic necrosis of bone with or without associated soft tissue necrosis of variable extent, occurring in the absence of local primary tumor necrosis, recurrence, or metastatic disease [33]. The reported prevalence of ORNJ is ~3–7% [34, 35]. The mandible, radiation doses ≥ 65–70 Gy [25, 26], co-morbidities (e.g., diabetes mellitus, excessive alcohol consumption), poor oral health, invasive dental treatment and ill-fitting prosthesis have been associated with higher risk of ORNJ development [24, 27–32, 36]. The treatment of ORNJ is based on the severity and remains challenging. Current treatment modalities range from antibiotic therapy, combination therapy with pentoxifylline, tocopherol and/or clodronate, hyperbaric oxygen therapy and surgical intervention [37–39]. Other significant oral manifestations arising from HNRT include permanent salivary gland hypofunction and trismus which can occur at radiation doses as low as 20 and 50 Gy, respectively [40–43]. Both conditions exponentially increase the patient's caries risk resulting in rapidly progressing dental decay. In view of the life-long risk of ORNJ and its associated treatment challenges, the main objective of dental evaluation for HNRT patients is to eradicate local risk factors to minimize ORNJ risk. A secondary objective is to provide anticipatory guidance regarding preventive oral care strategies because of the high risk of rapidly progressing dental caries in post-HNRT patients.

### Anti-resorptive and Anti-angiogenic Therapy

The first reports of osteonecrosis of the jaw associated with bisphosphonates emerged in the early 2000s and was termed Bisphosphonates-Related Osteonecrosis of the Jaw [44]. This term was changed in 2014 to Medication Related Osteonecrosis of the Jaw (MRONJ) when reports of osteonecrosis of the jaw associated with the use of other anti-resorptive agents (ARAs) and anti-angiogenic agents (AAAs) were published [45]. ARAs are used in cancer therapy to prevent skeletal related events (e.g., pathological fractures, hypercalcemia of malignancy), while AAAs disrupt (neo) angiogenesis which hampers tumor growth and development. MRONJ is defined clinically by 3 criteria: (1) current or previous treatment with ARAs or AAAs; (2) exposed bone or bone that can be probed through an intra-oral or extra-oral fistula(s) in the maxillofacial region that has persisted for more than 8 weeks; and (3) no history of radiation therapy or obvious metastatic disease to the jaws [45]. The prevalence of MRONJ in cancer patients on ARAs or AAAs ranges widely between 0 and 18% [45, 46]. Longer duration of therapy, pre-existing inflammatory dental disease (e.g., periodontal disease), ill-fitting dentures, invasive dental procedures, uncontrolled diabetes mellitus, immunocompromised states and tobacco use are associated with higher risk [45–48]. Currently, there is no universally accepted treatment for MRONJ [46, 49, 50]. Treatment options include conservative symptomatic management, pharmacological interventions with pentoxifylline and tocopherol, hyperbaric oxygen therapy or surgical management [46, 49–51]. With the increasing use of ARAs and AAAs for cancer treatment, dental evaluation prior to the initiation of AAA or ARA therapies to address and mitigate modifiable risk factors associated with MRONJ development is considered routine in many cancer centers [45, 49].

## General Principles of Dental Evaluation Prior to Anti-Neoplastic Therapy

### Clinical Assessment

Thorough medical, dental and social histories as well as patient's dental complaints should be elicited as part of the clinical assessment prior to initiating anti-neoplastic therapy.

A comprehensive clinical examination begins with a thorough assessment of the extra-oral structures to evaluate for any sources of pain or infection. Next, the intraoral examination should include a systematic assessment of the oral mucosal tissues for soft tissue pathologies, opportunistic infections or other abnormalities. This should be followed by the assessment of the teeth for caries and quality of existing restorations. Teeth with large restorations or suspicious for pulpal or periapical pathologies should be further evaluated using adjunctive aids (e.g., pulp sensibility tests) to rule out acute and/or chronic infections. If present, oral prosthesis should be checked and adjusted for any areas that could cause mucosal trauma. A periodontal examination to identify the presence of deep or suppurative periodontal pockets, inflamed gingiva, clinical attachment loss and furcal exposure should be performed [4, 45, 52–58].

For radiographic examination, acquiring a dental panoramic pantogram (DPT) provides an overview of the general oral health status and is useful for identifying pathology (e.g., impacted teeth, cysts) [59, 60]. A baseline DPT should be taken if one is not available within the year, or if there is a clinical suspicion of an intra-bony pathology. Bitewing radiographs should be taken to assess for caries and to check the quality of existing restorations (i.e., recurrent caries) [59, 60]. For patients with bitewings that were done within a year, a new set of bitewing radiographs may not be needed if the suspicion for new caries is low. Periapical radiographs should be captured for both asymptomatic and symptomatic teeth with large cavities and restorations to rule out pulpal or periapical pathologies as well as to assess the periodontal health [59, 60].

### Treatment Planning

#### Considerations

Treatment planning is directed by the nature and urgency of the dental problem, the time available to complete the treatment, the patient's medical fitness and considerations unique to the type of anti-neoplastic therapy [2].

The dental practitioner should consider the potential for a dental finding to develop into an infection or become a problem in the future, and the consequences of treatment versus no treatment. The considerations differ based on the type of anti-neoplastic therapy planned which has been discussed in the earlier section.

Another consideration is to prioritize and sequence dental procedures to ensure sufficient time for healing. For example, dental extractions should be performed earlier to allow time for wound mucosalization. Typically, the minimum healing durations prior to initiation of chemotherapy and HNRT/ARA/AAA therapies are ~7–10 days and 10–14 days, respectively [45, 56, 61, 62].

For cancer patients who are immunosuppressed from their underlying illness or as a consequence of their anti-neoplastic therapy, a baseline complete blood count may be necessary to assess the need for antibiotic prophylaxis or blood transfusions prior to invasive dental procedures [63]. Although recommendations may vary across different centers, the common thresholds to determine the need for antibiotic prophylaxis and platelet transfusions are absolute neutrophil count 1 × 10^9^/L (<1000/mm^3^) and platelet count of 60 × 10^9^/L (<60,000/mm^3^), respectively [35, 64]. Another consideration for necessitating antibiotic prophylaxis is the presence of a central indwelling catheter because of the potential for a distant site infection after an invasive dental procedure. However, evidence supporting this practice is limited [65].

For patients undergoing high dose HNRT, the advent of Intensity Modulated Radiation Therapy (IMRT) has allowed for continued high dose delivery to the tumor bed while reducing the radiation to the adjacent tissues [66–68]. This has resulted in some reduction of the oral toxicities induced by HNRT [28, 69]. Polce et al. had further explored using the IMRT radiation plans to estimate the radiation dose to each tooth or selected area of interest so that decision making during treatment planning can be more precise [66]. Other local measures include fabrication of intra-oral stents to be worn during HNRT treatment sessions to decrease radiation scatter in patients with heavily restored dentition, to displace the tongue or to position the oral structures away from the epicenter where the radiation dose is at the highest [70, 71]. While potentially effective in reducing the oral side effects of HNRT, intra-oral stents are not widely used due to the lack of standardized protocols and limited high-quality evidence [71, 72]. Patients also often find the intra-oral stents bulky and uncomfortable, especially for those experiencing oral pain and trismus [72].

Lastly, while it is ideal to eliminate all dental disease, the clinician must consider the intent of the anti-neoplastic therapy during the treatment planning process. The benefit of total dental disease eradication in patients undergoing palliative treatment should be balanced against the discomfort and post-operative sequelae of extensive dental procedures.

#### Dental Clearance Protocols

Conventionally, the objective of dental clearance has been to eliminate all dental pathology prior to anti-neoplastic therapy. However, the complete clearance approach may carry some risk of complications arising from the dental treatment itself [2, 73–75]. Tai et al. reported that 40% of patients who had third molar extractions prior to their anti-neoplastic therapy developed post-operative complications (e.g., alveolar osteitis) [76]. Another consideration is when there is inadequate time to complete all planned treatment, and for treatment to be completed and adequate healing to occur, anti-neoplastic therapy would have to be delayed. This is not ideal because of the well-documented association between delay in anti-neoplastic therapy initiation and poorer survival rates [77].

In recent years, the concept of partial or minimal dental clearance protocols have emerged in the literature [2]. A partial clearance protocol allows for a less aggressive dental clearance and does not require for all dental pathologies to be eliminated prior to the anti-neoplastic therapy. A minimal protocol involves the treatment of only symptomatic oral disease. In a systematic review evaluating the adequacy of the partial and minimal dental clearance protocols prior to chemotherapy and HSCT, the authors recommended that a partial dental clearance protocol may be appropriate when there is insufficient time for complete dental clearance [2]. However, whenever possible, complete treatment clearance protocol is preferred [2]. Table 1 provides an overview of the typical procedures performed in complete and partial clearance protocol [52, 58, 73, 78–85].

**Table 1:** Summary of complete and partial dental clearance protocols [52, 58, 73, 78–85].

	**Complete clearance protocol**	**Partial clearance protocol**
Caries prevention	•Application of professional topical fluoride varnish at least twice yearly	
	•Consider regular use of high fluoride (≥2,800 ppm) toothpaste	
Dental caries	•Extract non-restorable teeth, teeth with guarded or poor prognosis and retained roots	•Treat only large or symptomatic carious teeth
	•Restore all carious teeth	•Restore teeth with mild and moderate caries only if time permits. If not, regular topical fluoride therapy application is advised. Silver diamine fluoride may also be considered
	•Replace all defective restorations	•Treat only defective restorations that are symptomatic
Non carious lesions	•Restore non-carious lesions that affect maintenance of good oral hygiene •Extract large non-carious lesions that approximate the pulp	•Treat only symptomatic non-carious lesions
Pulpal and periapical pathology	•Extract primary teeth with deep caries, pulpal or periapical pathology •Permanent teeth - Symptomatic and asymptomatic non-vital teeth: Initiate root canal treatment at least 1 week before anti-neoplastic therapy to allow for sufficient time to assess treatment success. If not possible, extraction should be considered - Previously root canal treated teeth with apical periodontitis: Retreat, extract or perform apicoectormy	•Treat only symptomatic teeth with apical periodontitis and/or periapical lesion ≥ 5 mm
Periodontal disease	•Professional cleaning	
	•Extract teeth with advanced periodontal disease (probing depth ≥ 6 mm, furcation I, II, III, tooth mobility II-III)	•Extract only teeth with severe periodontal disease (probing depth ≥ 8 mm, mobility III)
Prosthesis and	•Check dentures for irregularities or sharp edges and adjust accordingly	
appliances	•Remove orthodontic appliances that may aggravate mucosal injury	
	•Modify, disassemble or replace fixed prosthesis suspicious of recurrent caries, marginal leakage or affecting maintenance of good oral hygiene	•Modify, disassemble or replace only fixed prosthesis with large or symptomatic caries
Misaligned teeth	•Extract supra-erupted and grossly misaligned teeth	•No recommendation
Exfoliating teeth	•Extract mobile deciduous teeth with >50% physiological root resorption or those that are expected to exfoliate	•Extract only severely mobile deciduous teeth that are expected to exfoliate within a few weeks
Partially impacted third molars	•Extract asymptomatic and symptomatic partially erupted impacted third molars	•Extract only partially erupted impacted third molars with evidence of pericoronitis or purulence

### Delivery of Dental Treatment

Dental evaluation and treatment should be ideally performed prior to the initiation of anti-neoplastic therapy. If extractions are required after HNRT, some authors have suggested that atraumatic extractions are best performed within 6 months after HNRT to mitigate the risks of ORNJ [86–88]. This recommendation is based on a landmark histology study by Marx et al. whereby serial biopsies from 64 patients at varying times (unspecified) during and after receiving 72 Gy of HNRT demonstrated hyperemia and endarteritis in the first 6 months post-HNRT [74]. After which, the tissues demonstrated hypovascularity and fibrosis that progressively worsened with time [74]. In a recent systematic review evaluating the incidence of ORNJ in patients who had dental extractions before or after HNRT, authors found no difference in ORNJ incidence between the 2 groups [89]. However, authors cautioned that these results were based on vastly heterogeneous studies that lacked detail regarding the timing of dental procedures in relation to HNRT and recommended the need for larger longitudinal studies [89].

### Patient Education

**Figure 1:**
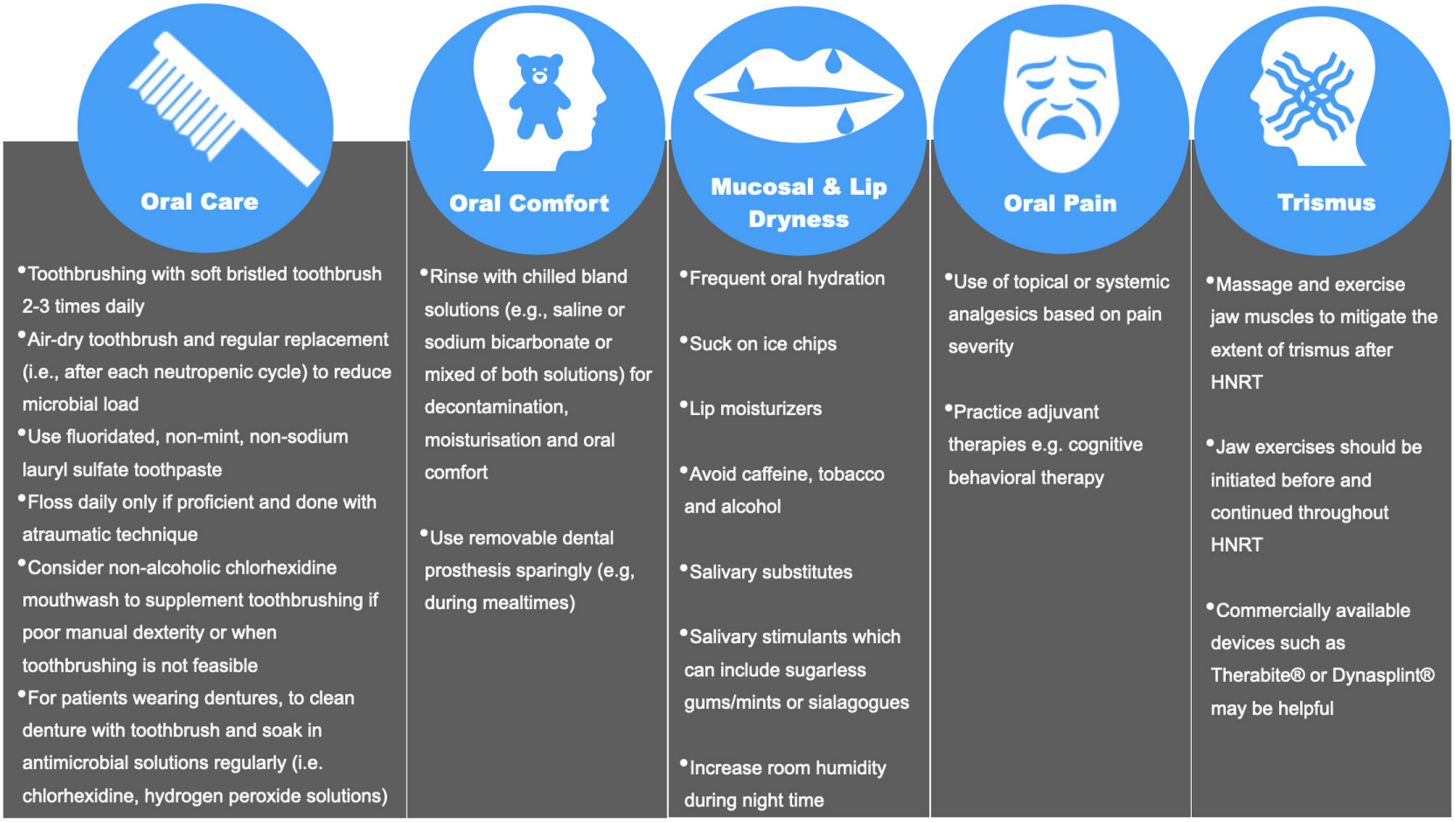
Oral care instructions during and after anti-neoplastic therapy [54, 63, 92–101].

Patient education is an essential element of the dental clearance protocol. The dental professional should communicate with the patient about the rationale for dental evaluation, the potential acute and chronic oral complications and the recommended oral care during anti-neoplastic therapy (Figure 1) [54, 63, 90–101]. The recommendations should be customized to the patient's needs, which is dependent on the type of anti-neoplastic therapy as well as their underlying medical and dental conditions.

## Oral Care During and After Anti-Neoplastic Therapy

The objectives of oral care during and immediately after anti-neoplastic therapy are to prevent infections, control pain, maintain function and manage acute and chronic oral complications [4].

During active anti-neoplastic therapy, elective dental treatment should be avoided. In the event of an acute dental infection, pharmacological intervention with antibiotic therapy and analgesics are the preferred management modality [83]. If an emergency dental procedure is required (e.g., severe odontogenic abscess with potential airway embarrassment), the dental practitioner should plan for dental treatment in liaison with the patient's oncologist or medical physician. Specific pre-procedure considerations include the need for antibiotic prophylaxis, replacement of blood products and in some situations, disruption of anti-neoplastic therapy.

After active anti-neoplastic therapy or in patients with a history of cancer, 3–6 monthly routine reviews are recommended, and the interval is based on patient's dental needs. Other than addressing patient's complaints and performing a comprehensive clinical examination at these reviews, the dental professional should carefully evaluate the oral cavity for signs or symptoms of chronic oral manifestations from anti-neoplastic therapies as well as recurrence and occurrence of secondary malignancies. At the review, the importance of maintaining a good oral hygiene homecare program should also be reiterated.

## Conclusion

Dental clearance prior to anti-neoplastic therapy is routine in many cancer centers. To be able to deliver the best care for the patient, it is essential for the dental practitioner to be aware of the rationale and objectives for dental evaluation as well as the specific considerations unique to the various anti-neoplastic treatment modalities.

## Author Contributions

CWY contributed to majority of the writing. AR contributed to the framework of the manuscript, provided expertise in the area, and checked the manuscript for accuracy. CH developed the framework for the manuscript and contributed to the writing. All authors contributed to the article and approved the submitted version.

## Conflict of Interest

The authors declare that the research was conducted in the absence of any commercial or financial relationships that could be construed as a potential conflict of interest.

## Publisher's Note

All claims expressed in this article are solely those of the authors and do not necessarily represent those of their affiliated organizations, or those of the publisher, the editors and the reviewers. Any product that may be evaluated in this article, or claim that may be made by its manufacturer, is not guaranteed or endorsed by the publisher.
